# Honokiol, a Lignan Biphenol Derived from the Magnolia Tree, Inhibits Dengue Virus Type 2 Infection

**DOI:** 10.3390/v7092852

**Published:** 2015-09-10

**Authors:** Chih-Yeu Fang, Siang-Jyun Chen, Huey-Nan Wu, Yueh-Hsin Ping, Ching-Yen Lin, David Shiuan, Chi-Long Chen, Ying-Ray Lee, Kao-Jean Huang

**Affiliations:** 1 Department of Pathology, Wan Fang Hospital, Taipei Medical University, Taipei 116, Taiwan; phildts@gmail.com (C.-Y.F.); chencl@tmu.edu.tw (C.-L.C.); 2 Department of Life Science and Institute of Biotechnology, National Dong Hwa University, Hualien 974, Taiwan; 610013122@ems.ndhu.edu.tw (S.-J.C.); jouyuan22@gmail.com (C.-Y.L.); shiuan@mail.ndhu.edu.tw (D.S.); 3 Institute of Molecular Biology, Academia Sinica, Taipei 11529, Taiwan; hnwu@gate.sinica.edu.tw; 4 Department and Institute of Pharmacology, School of Medicine, National Yang-Ming University, Taipei 112, Taiwan; yhping@ym.edu.tw; 5 Institute of Biophotonics, National Yang-Ming University, Taipei 112, Taiwan; 6 Department of Pathology, Taipei Medical University Hospital, Taipei Medical University, Taipei 110, Taiwan; 7 Department of Medical Research, Chiayi Christian Hospital, Chiayi 600, Taiwan; 8 Department of Nursing, Min-Hwei College of Health Care Management, Tainan 73658, Taiwan; 9 Institute of Biologics, Development Center for Biotechnology, New Taipei City 22180, Taiwan

**Keywords:** antiviral, honokiol, dengue virus, *Magnolia*

## Abstract

Dengue is the most widespread arbovirus infection and poses a serious health and economic issue in tropical and subtropical countries. Currently no licensed vaccine or compounds can be used to prevent or manage the severity of dengue virus (DENV) infection. Honokiol, a lignan biphenol derived from the *Magnolia* tree, is commonly used in Eastern medicine. Here we report that honokiol has profound antiviral activity against serotype 2 DENV (DENV-2). In addition to inhibiting the intracellular DENV-2 replicon, honokiol was shown to suppress the replication of DENV-2 in baby hamster kidney (BHK) and human hepatocarcinoma Huh7 cells. At the maximum non-toxic dose of honokiol treatment, the production of infectious DENV particles was reduced >90% in BHK and Huh7 cells. The underlying mechanisms revealed that the expression of DENV-2 nonstructural protein NS1/NS3 and its replicating intermediate, double-strand RNA, was dramatically reduced by honokiol treatment. Honokiol has no effect on the expression of DENV putative receptors, but may interfere with the endocytosis of DENV-2 by abrogating the co-localization of DENV envelope glycoprotein and the early endosomes. These results indicate that honokiol inhibits the replication, viral gene expression, and endocytotic process of DENV-2, making it a promising agent for chemotherapy of DENV infection.

## 1. Introduction

Dengue fever is an acute infectious disease caused by the dengue virus (DENV), an enveloped, positive-sense, single stranded RNA virus belonging to the *Flaviviridae* family. There are four definite serotypes of DENV (DENV-1, -2, -3, and -4) and an emerging new one has recently been reported [1]. Dengue is transmitted by mosquito vectors, principally *Aedes aegypti* and *Aedes albopictus*. It is characterized by biphasic fever, headache, pain, rash, lymphadenopathy, and leucopenia [2,3]. In most cases, the disease of dengue fever is self-limited. However, there is a risk of progressing to severe dengue hemorrhagic fever (DHF) or dengue shock syndrome (DSS), especially when cross infection of different serotypes of DENV occurs. Under such conditions, bleeding, low levels of blood platelets and blood pressure, and blood plasma leakage occur, which leads to a critical or even life-threatening situation of affected patients. Approximately 3.6 billion people living in tropical and subtropical areas are affected by dengue viruses and over 100 million infections are reported annually [4]. Dengue has become an enormous health and economic concern in these endemic countries.

Phytochemicals, which occur naturally in plants, have been a major subject in study for their health benefits and potential pharmaceutical applications. Many phytochemicals are found to have versatile effects, including antioxidant, anticancer, anti-inflammatory, anti-bacterial, and anti-viral effects [5,6,7,8]. Viruses, unlike bacterial pathogens, only exhibit biological activities when they infect host cells and utilize host cell mechanisms to replicate within them. The search for active phytochemicals against pathogenic viruses without having deleterious effects on the host has been the focus of studies in recent decades. The antiviral mechanism of these agents may be ascribed to their antioxidant/scavenging capacities, nucleic acid synthesis inhibition, viral entry suppression, or inhibition of the viral reproduction [7].

**Figure 1:**
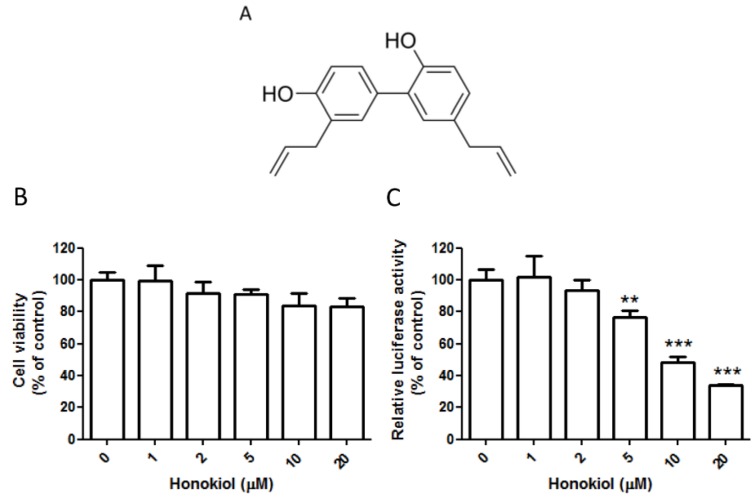
Honokiol inhibits DENV (dengue virus) replicon. (**A**) The chemical structure of honokiol; (**B**) BHK (baby hamster kidney) cells were treated with various concentrations of honokiol (1–20 μM) for 24 h, and the viability of BHK cells was measured by MTT assay; (**C**) The luciferase activity was measured in cell lysates after treatment with honokiol (1–20 μM) for 24 h. Data indicate the average value of triplicates (mean ± SD). ** *p* = 0.006; *** *p* < 0.001, as compared with the vehicle control.

Honokiol is a lignan biphenol derived from the *Magnolia* tree (Figure 1A). The bark or seed cones of the tree, such as in the Chinese herbal medicine *Hou-Pu* (derived from *Magnolia officinalis*), have been widely used in traditional medicine as analgesic, distension, or anxiety relief [9]. Honokiol is a pleiotropic compound that has a variety of pharmacological effects, including antitumorigenic [10], anti-inflammation [11], anti-thrombosis [12], anti-oxidation [13], and neuroprotective effects [14]. Being a relatively small molecule, it has been proved to be able to readily cross the blood-brain barrier and the blood-cerebrospinal fluid barrier [15]. Recently, it has been reported that honokiol can suppress the infection of hepatitis C virus in cell models by targeting viral entry and replication [16]. As a result, honokiol is a potential pharmaceutical agent with high bioavailability.

Currently there is no licensed vaccine for dengue, although a live attenuated chimeric vaccine is under Phase III trial [17]. The prevention of dengue fever to date is solely mounted by vector control. In addition, no anti-dengue viral agent is clinically available to treat or reduce the severity of dengue symptoms. Only supportive medical treatment is applied to patients with dengue diseases. Although DHF and DSS occur in less than 5% of all cases of dengue [18,19], severe cases that result in death are predominant in children under the age of 15 [20]. Considering the severity of dengue diseases, it is vital and urgent to develop strategies to combat DENV infection. Several phytochemicals have been reported previously to have antiviral activities [7]. Here we report that honokiol has a significant anti-DENV activity *in vitro*. Treatment of honokiol reduces the activity of DENV-2 replicon and suppresses the viral replication. After DENV infection, honokiol treatment suppresses the expression of viral protein NS1 and NS3 and the viral replicating intermediate, double-strand RNA (dsRNA). Honokiol may also interfere with the endocytotic process during DENV entry. These results support that honokiol intervenes in DENV-2 infection at multiple stages, making it a potent anti-DENV chemical for clinical dengue treatment.

## 2. Results

### 2.1. Honokiol Effectively Inhibits the DENV Replicon

A stably transfected baby hamster kidney (BHK) cell line, BHK-D2-Fluc-SGR-Neo-1, which harbors a luciferase-reporting DENV-2 subgenomic replicon [21], was used to determine the inhibitory effect of honokiol on DENV-2 replication. The cytotoxic effect of honokiol on this BHK-DENV cell line was evaluated by a standard MTT assay with various concentrations of honokiol (1–20 µM) treatment. At 24 h, the treatment of honokiol did not have a marked effect on the viability of this cell from 1–5 µM (Figure 1B). At 10 and 20 µM of honokiol treatment, a minor reduction of the viability of the BHK-DENV was observed, but the overall survival of cells was ≥80% compared to the vehicle control. Under these conditions, honokiol was found to inhibit the luciferase activity of DENV replicon in a dose-dependent manner (Figure 1C). At 5 µM, the reporter activity was decreased 23.3% ± 3.3 compared to the vehicle controls (*p* = 0.006). The reduction of luciferase activity was even more prominent at 10 and 20 µM of honokiol treatment (51.6% ± 2.5 and 65.1% ± 0.6 reduction, respectively; *p* < 0.001) with a minor effect on cell viability. This result indicates that honokiol has a significant antiviral activity against DENV-2 replicon intracellularly.

### 2.2. Honokiol Inhibits the DENV Infection *in Vitro*

**Figure 2:**
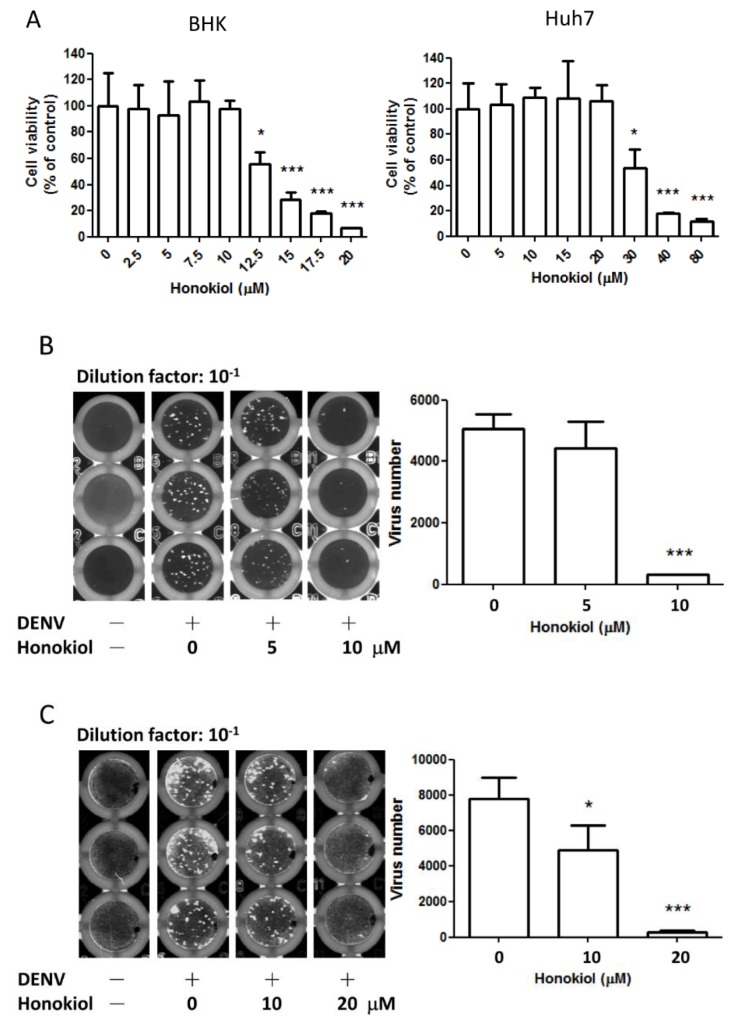
Honokiol decreases dengue virus production. (**A**) The cytotoxicity of honokiol on BHK and Huh7 cells was measured by MTT assay. Various concentrations of honokiol were applied to cells for 48 h; (**B**,**C**) Infectious DENV-2 particles released from DENV-infected, mock/honokiol-treated (**B**) BHK and (**C**) Huh7 cells were determined by fluorescence focus assay. Quantification of the virus number was calculated by (fluorescence focus units) × (dilution factor) × (total supernatant volume) and plotted as a bar chart. Data indicate the average value of triplicates (mean ± SD). * *p* < 0.05; *** *p* < 0.001, as compared with the control.

The DENV replicon test had indicated that honokiol has a substantial antiviral activity against DENV. To further confirm this observation, an *in vitro* DENV yield reduction assay was performed. Two cell lines, the BHK cell and the human hepatocarcinoma cell Huh7, were selected for the viral yield reduction assay. The cytotoxic effect at 48 h after honokiol treatment was first determined by MTT assay (Figure 2A). The half maximal cytotoxic concentration (CC_50_) of honokiol was found to be 13.35 ± 1.13 µM for BHK cells and 31.19 ± 1.49 µM for Huh7 cells. While at 10 and 20 µM of honokiol treatment, no deleterious effect was observed in BHK and Huh7 cells, respectively, and therefore, that concentration was selected as the maximum non-toxic dose (MNTD) for each cell line in the following studies. The BHK and Huh7 cells were infected with DENV-2 and then followed by honokiol treatment with different concentrations for 48 h. The released infectious DENV particles in the cell culture supernatant post honokiol treatment was determined by fluorescence focus assay. Treatment of honokiol was found to suppress the viral production both in DENV-infected BHK and Huh7 cells (Figure 2B,C). In BHK cells, treatment with 5 µM of honokiol did not reveal a marked reduction, while 10 µM of honokiol significantly reduced the DENV production (*p* < 0.001, Figure 2B). In Huh7 cells, the inhibition of DENV production was significant at 10 µM of honokiol treatment (37% reduction, *p* = 0.027), and was reduced further at 20 µM (*p* < 0.001, Figure 2C). The reduction of virus production was >90% at the MNTD (10 µM and 20 µM, respectively) of honokiol in both BHK and Huh7 cells. These results show the profound DENV inhibition potency of honokiol in decreasing the massive viral yield.

### 2.3. Honokiol Inhibits DENV Protein Expression and Viral RNA Replication

**Figure 3:**
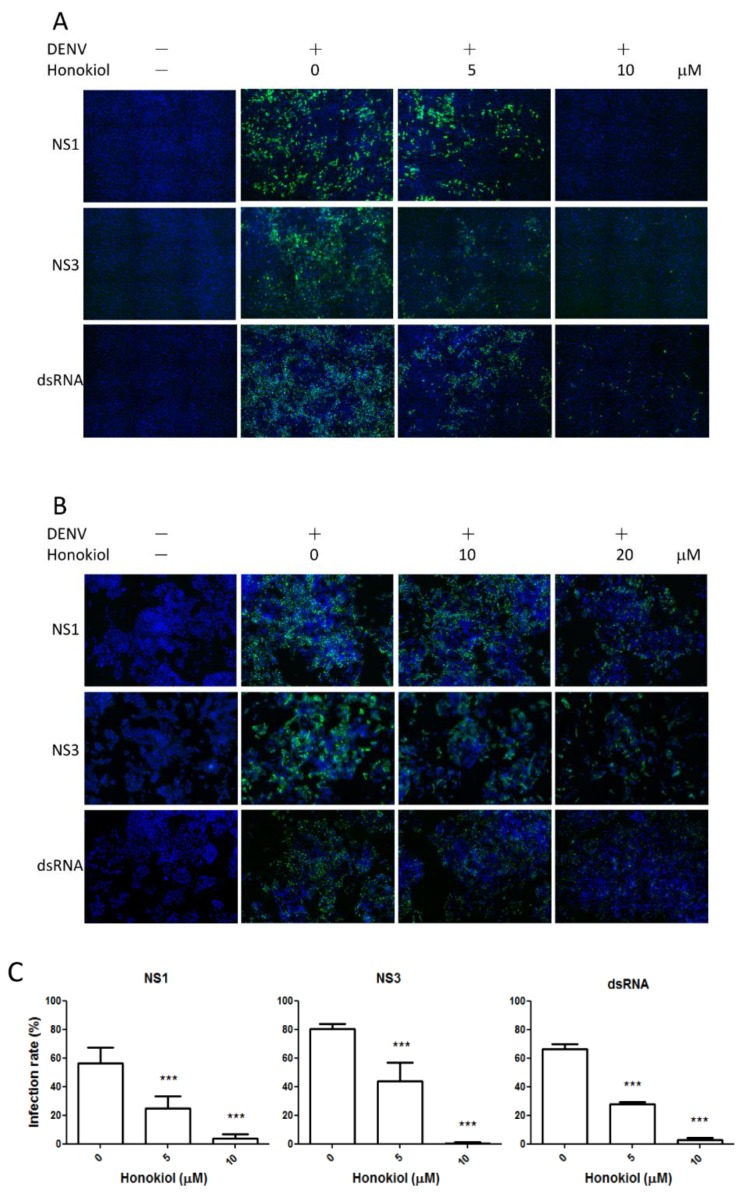

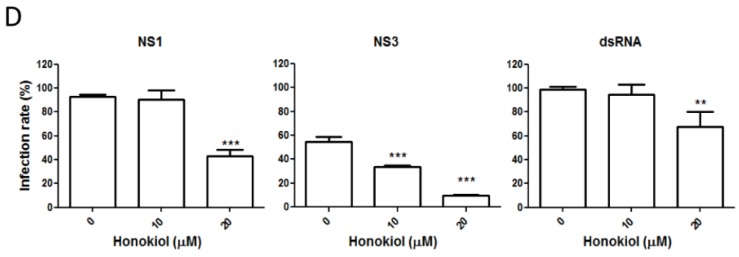
Honokiol inhibits dengue virus translation and replication in cells. (**A**) Immunofluorescence assay of honokiol-treated, DENV-infected BHK cells with MOI = 0.1. The viral NS1, NS3, and dsRNA antigens were detected after 48 h of honokiol treatment; (**B**) Immunofluorescence assay of honokiol-treated, DENV-infected Huh7 cells with MOI = 10. The viral NS1, NS3, and dsRNA antigens were detected after 48 h of honokiol treatment; (**C**, **D**) The percentage of viral NS1, NS3, and dsRNA positive cells was analyzed using the BD Pathway^TM^ 435 Bioimaging system in (**C**) BHK and (**D**) Huh7, respectively. (Graph is plotted through calculating the percentage of NS1, NS3, and dsRNA expression in each image.) Data indicate the average value of triplicates (mean ± SD). ** *p* = 0.01; *** *p* < 0.001, as compared with the control.

To investigate if honokiol could inhibit the viral protein expression as well as the viral RNA replication, BHK and Huh7 cells were infected with DENV-2 and then treated with honokiol for 48 h. The expression levels of the viral non-structure protein NS1 and NS3, and the viral replicating intermediate, double-strand RNA (dsRNA), were assayed using immunofluorescence staining and analyzed by high content image analysis. In both DENV-infected BHK and Huh7 cells, the cells positive for NS1, NS3 or dsRNA were decreased after honokiol treatment, indicating their expressions were suppressed (Figure 3A,B). The percentage of DENV-infected cells positive for the viral antigens (*i.e.*, NS1, NS3 or dsRNA) in each experiment was quantified by the Attovision software (BD Biosciences) and the results were plotted and statistically evaluated (Figure 3C,D). In the BHK cell model infected with DENV-2, the percentage of cells with the NS1, NS3 or dsRNA signal was reduced to near or below 50% by 5 µM of honokiol treatment. At 10 µM, the signal of these three viral products had almost completely vanished (reduction >90%, *p* < 0.001, Figure 3C). The inhibitory effect was less intense in Huh7 compared to BHK cells. At 10 µM of honokiol treatment, the viral NS3 expression showed a sensitive response to honokiol-mediated inhibition (*p* < 0.001, Figure 3D) as compared to the NS1 and dsRNA expression. However, a significant reduction of these three viral products was observed at 20 µM of honokiol treatment (*p* = 0.01 for dsRNA; *p* < 0.001 for NS1 and NS3). These results indicate that treatment of honokiol after DENV infection can not only inhibit the DENV protein translation (*i.e.*, NS1 and NS3 expression) but also suppress its subsequent RNA replication (*i.e.*, dsRNA expression), which may lead to the decreased viral production observed in previous viral yield assay (Figure 2B,C).

### 2.4. Pretreatment with Honokiol Does Not Affect the Attachment of DENV on Host Cells

**Figure 4:**
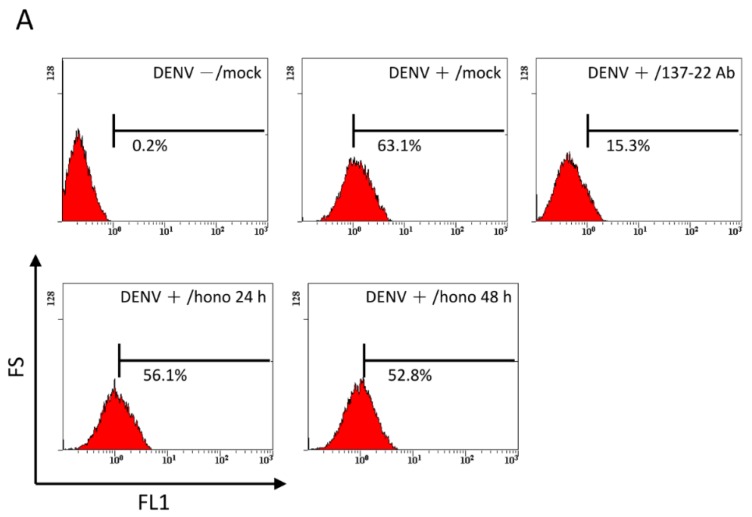

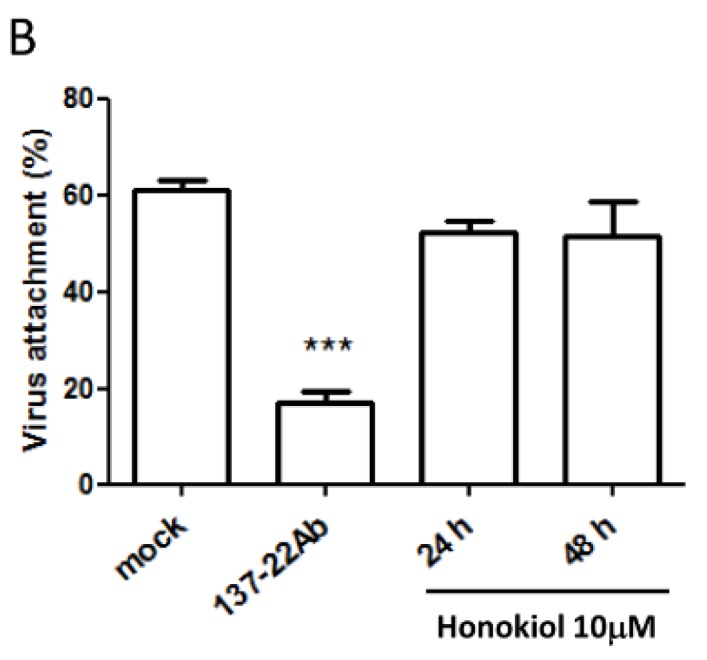
Pre-treatment of honokiol does not affect DENV receptor expression in cells. (**A**) BHK cells were treated with honokiol at 10 μM concentration for 24 or 48 h and then incubated with dengue virus for 30 min to process the virus attachment. Simultaneously treatment of neutralizing antibodies 137-22 upon infection was used as a control group to block the specific DENV binding. Unbound virus was washed away with PBS and the cells were labeled with anti-DENV E protein antibody and secondary antibody for flow cytomerty analysis; (**B**) The percentage of cells showing positive fluorescent signals was plotted. Data indicate the average value of triplicates (mean ± SD). *** *p* < 0.001 as compared with the control.

Attachment is the first step of virus entry. It is mediated by virion proteins binding to specific surface receptors such as membrane proteins, lipids, or the carbohydrate moieties present on glycoproteins or glycolipids [22,23]. To investigate if pre-treatment of honokiol inhibits the attachment of DENV to host cells, the BHK cells were first treated with 10 µM of honokiol for 24 and 48 h prior to infection with DENV. The flow cytometry analysis showed a marked increase of fluorescence signal in BHK cells when incubated with DENV-2, confirming the attachment of DENV to the host cells (Figure 4A,B). As expected, the presence of neutralizing antibodies 137-22 upon infection significantly reduced the fluorescence signal. Pre-treatment of honokiol did not alter the degree of DENV binding to the cell surface, regardless of treatment times. This result indicates that honokiol-mediated DENV inhibition is not related to affect the expression of DENV receptor(s) on host cells.

### 2.5. Honokiol May Interfere with the Endocytic Pathway during DENV Entry

**Figure 5:**
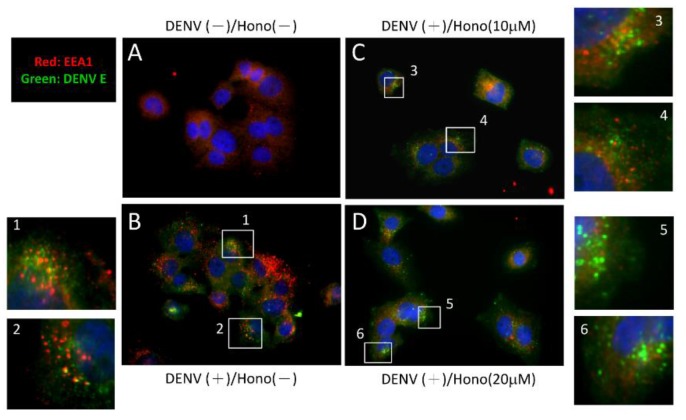
Honokiol may interfere with the endocytotic pathways of DENV entry. Huh7 cells were (**A**) mock or (**B**) infected with dengue virus at MOI of 10. The DENV-infected cells were treated with (**C**) 10 and (**D**) 20 μM of honokiol for 1.5 h. Immunofluorescence staining was conducted to detect the DENV E protein (green fluorescence) and the early endosome marker, EEA1 (red fluorescence). Numbered side squares represent the magnified images of areas in corresponding panels.

To further characterize the antiviral mechanisms of honokiol, we investigated the effect of the compound on the entry of the DENV particle into host cells. Following attachment, the DENV is internalized via receptor-mediated endocytosis [24]. Upon binding to receptors, the clathrin-mediated endocytosis is considered to be a major pathway for DENV entry [24]. In this study, honokiol was applied to Huh7 cells after DENV attachment to test if it can interfere with the viral entry process. Infection with DENV increased the amount of intracellular early endosomes (Figure 5A,B), and the DENV E protein was found to co-localize with the early endosomes in DENV-infected cells, indicating its interactions, which is a distinct early event of endocytosis during DENV entry [25,26]. Intriguingly, the presence of honokiol (10 and 20 µM) after DENV entry substantially suppressed the up-regulation of early endosomes, as compared to the DENV infected/mock-treated cells (Figure 5C,D *vs.* 5B). Consequently, the co-localization of DENV E protein and the early endosome was markedly abrogated. This result indicates that honokiol may interfere with the endocytotic process of DENV during its entry into the host cells.

### 2.6. Honokiol Mediated DENV Inhibition is Not Attributed to the Influence on Cell Cycle Progression

**Figure 6:**
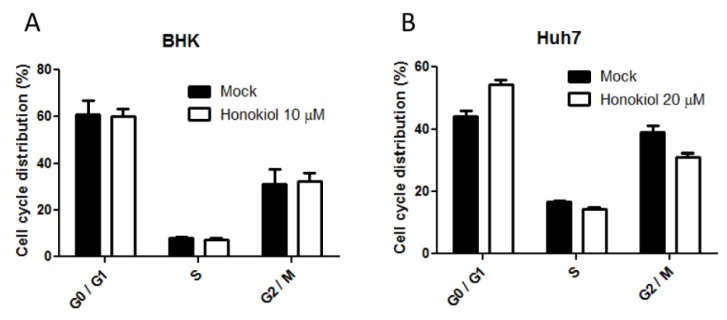
Honokiol induces a slight cell cycle alteration in Huh7 but not BHK cells. (**A**) BHK and, (**B**) Huh7 cells were mock or treated with honokiol for 48 h. The cells were then fixed, stained with PI and analyzed by flow cytomerty. The distribution of cell cycle phases was demonstrated by bar charts. Data indicate the average value of triplicates (mean ± SD).

Studies have demonstrated that honokiol can induce G0-G1 cell cycle arrest in cancer cells [27]. In a previous study, hepatocarcinoma cell HepG2 was demonstrated to be more permissive for DENV infection and virus production in the G2 phase as compared to other phases examined [28]. Although the concentration of honokiol used in this study was equal or lower than the maximum non-toxic dose for cells, there is a possibility that the cell cycle modulation by honokiol may affect the propagation of DENV. In this study, the effect of honokiol on cell cycle regulation was examined. In BHK cells, treatment of 10 µM honokiol for 48 h did not result in any alteration of cell cycle progression (Figure 6A and Figure S1A). In Huh7 cells, a slight increase of G0/G1 and decrease of G2 phase was observed after 48 h of 20 µM honokiol treatment (Figure 6B and Figure S1B). However, compared to the profound inhibition of DENV propagation at 10 and 20 µM of honokiol treatment in BHK and Huh7 cells, respectively (Figure 2B,C), the influence of cell cycle modulation by honokiol in DENV production is insignificant. This result suggests that the inhibition of DENV propagation by honokiol is not mediated mainly through altering the cell cycle phases in BHK and Huh7 cells.

### 2.7. Neither NF-κB Activation Nor IFN-β Expression was Essential in Honokiol Mediated DENV Inhibition

**Figure 7:**
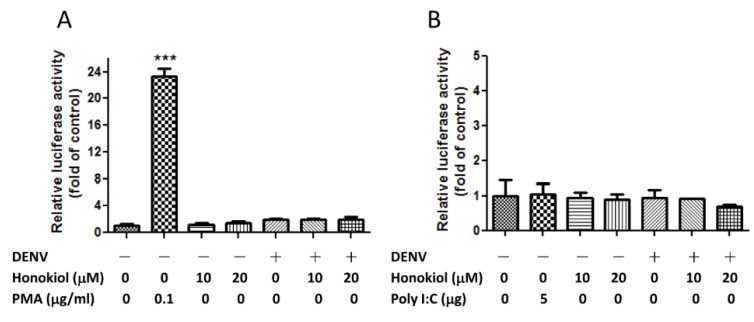
Honokiol does not alter the activation of NF-κB and the expression of IFN-β. (**A**) Assay of NF-κB activation. Huh7 cells transfected with NF-κB-luciferase reporters were mock or infected with DENV at MOI = 5 and then treated with honokiol at 10 and 20 μM for 48 h, followed by Firefly-Renilla luciferase assay to determine the relative expression of NF-κB responsive luciferase. PMA is an NF-κB activator and was used as a positive control; (**B**) Assay of IFN-β promoter activity. Huh7 cells transfected with IFN-β-luciferase reporters were mock or infected with DENV at MOI = 5 and then treated with honokiol at 10 and 20 μM for 48 h, followed by Firefly-Renilla luciferase assay to determine the relative expression of IFN-β promoter-driven luciferase. Poly(I:C) is a immunostimulant similar to dsRNA and was used as a control. Data indicate the average value of triplicates (mean ± SD). *** *p* < 0.001 as compared with the control.

Nuclear factor-kappa B (NF-κB) is an essential molecule in the induction of type I interferon beta (IFN-β) and cytokines in the toll-like receptor (TLR) signaling pathway [29,30]. The strain of DENV-2 PL046 used in this study has been shown to trigger a low degree of NF-κB p65 nuclear translocation [31]. Thus, the DENV inhibition by honokiol was checked to see if the NF-κB activation was involved with the use of an NF-κB-dependent luciferase reporter. It was found that the infection of DENV in Huh7 cells did not induce the activation of NF-κB, a phenomenon also observed in a previous study [32], nor did the treatment with honokiol for 48 h in mock or DENV infection (Figure 7A). In fact, honokiol was reported to inhibit NF-κB activation in several cell types [33,34]. Type I interferon (IFN-α/β) plays an important role in the generation of antiviral immune responses [35,36]. The ability of DENV to inhibit IFN-α/β signaling in cells was recognized and attributed to several DENV proteins [37,38,39,40,41]. To investigate if honokiol up-regulates the IFN-β expression to reduce the DENV infectivity, an IFN-β promoter-reporter assay was conducted in Huh7 cells. Similarly, it was found that neither honokiol treatment nor DENV infection in Huh7 cells induced the expression of IFN-β (Figure 7B). Additionally, the nuclear translocation of interferon regulatory factor-3 (IRF-3), an upstream factor that regulates the expression of IFN-β, was not observed in either DENV infection or honokiol or poly(I:C) treatments (Figure S2; poly(I:C) is an immunostimulant similar to dsRNA). The low/no responsiveness of the Huh7 cell line to poly(I:C) treatment was reported due to the intrinsic defect in TLR3 expression [42]. These results collectively indicate that honokiol does not exert its interference of DENV infection via the NF-κB or IFN-β pathways in this study.

## 3. Discussion

From a global health perspective, dengue virus (DENV) is the most widespread arbovirus infection in the last decades [43]. Infection with DENV leads to approximately 500,000 severe life-threatening cases and over 20,000 deaths every year [44]. Aside from attempts to control the spread of the mosquito vectors and efforts to develop a vaccine against DENV infection [45], the development of antiviral drugs for treatment of dengue fever and prevention of severe complications is an urgent priority. In this study, we report that honokiol interferes with DENV infection and propagation at multiple stages. Our study demonstrates that honokiol may be a potent anti-DENV target for antiviral therapy of dengue.

Honokiol is a component in the commonly used Chinese medicine *Hou-pu* from the bark of the *Magnolia* tree. *Hou-pu* has been widely used as a folk remedy for gastrointestinal disorders, anxiety, analgesic, and other diseases in Eastern medicine [9]. Honokiol has recently been shown to inhibit hepatitis C virus infection *in vitro* [16]. Our present study is the first study to demonstrate that honokiol possesses anti-DENV-2 activity. It would be interesting to investigate if honokiol also has an antiviral effect against other *Flavivirus*, such as yellow fever virus, West Nile virus, and Japanese encephalitis virus. The pharmacokinetics of honokiol has been evaluated in mice by intraperitoneal injection. At a dose of 250 mg/kg, the plasma concentration was reported to reach 1000 µg/mL (approx. 3755 µM) between 20–30 min after administration [46]. The pharmacokinetics of honokiol by oral administration of *Hou-pu* extracts had also been studied in rats. At a dose of 5 g/kg extracts (which contained 1.61 mg/kg honokiol), the maximum plasma concentration of honokiol was reported to be 1.966 mg/L (approx. 7.5 µM) [47]. The relative small molecular weight of honokiol and its lipophilic characteristic likely contributes to its rapid distribution at multiple sites and beyond the blood-brain barrier [15]. In some severe DHF cases, encephalopathy is observed in patients with high morbidity and mortality [48]. Recent evidence has suggested that DENV is capable of central nervous system infection [49]. Since honokiol is able to readily cross the blood–brain barrier and the blood-cerebrospinal fluid barrier [15], it may serve as a potent agent for treatment of this critical complication.

The CC_50_ of honokiol in Huh7 cells is 31.19 µM at 48 h (Figure 2A), and the IC_50_ of honokiol (by reduction of DENV infection in fluorescence focus assay) in Huh7 cells is 10.6 µM (Figure 2C). The selective index of the drug, when calculated by CC_50_/IC_50_, is 2.94. This relative low ratio could be attributed to the apoptosis-inducing effects of honokiol on many tumor or transformed cells [10,27]. Honokiol had been demonstrated to selectively inhibit the proliferation of transformed cells while not affecting normal cells under the same condition. It has been reported that at 20 µM of honokiol treatment for 48 h, there is no cytocidal effect on human normal peripheral blood mononuclear cells while the inhibition of B leukemia cells is over 95% [50]. The CC_50_ of Huh7 hepatoma cells was found to be 31 µM in our study (Figure 2A) and 35 µM in another study [16] under honokiol treatment, while the CC_50_ of normal AML12 hepatocytes was reported to be >150 µM [51]. In another study, at concentrations up to 40 µg/mL (approx. 150 µM), honokiol had little cytotoxicity on human fibroblast cells and lymphocytes [46]. It is apparent that normal cells are much more resistant to the cytotoxic effect of honokiol than tumor cells. These results may indicate that the selective index of honokiol in anti-DENV activity would be more significant in normal cells than the results we obtained by the transformed cells. In this study, we showed that honokiol inhibits the activity of DENV-2 replicons and suppresses DENV-2 replication in BHK and Huh7 cells (Figure 1 and Figure 2). Honokiol treatment reveals a profound and dose-dependent inhibition of DENV-2 replication, with more than 90% reduction at the maximum non-toxic dose (10 and 20 µM) as compared with the vehicle control in the focus-forming assay (Figure 2). At these concentrations, the effect of honokiol on cell cycle progression of BHK and Huh7 cells was found to be minimal (Figure 6). These results indicate that the prominent antiviral activity of honokiol is not attributed to its cytotoxic effect on host cells. In addition, treatment with honokiol was revealed to suppress the synthesis of viral dsRNA, and NS1/NS3 proteins (Figure 3). In BHK cells, treatment with 5 μM of honokiol induced a ~50% reduction of DENV dsRNA, NS1, and NS3, while at 10 μM these three viral products had almost completely subsided (Figure 3A,C). At 20 µM of honokiol treatment, a significant suppression of DENV NS1/NS3, and dsRNA was also noted in Huh7 cells (Figure 3B,D). The decrease of viral dsRNA by honokiol may result in reduced DENV replication observed in viral yield reduction and focus-forming assay (Figure 2B,C). It is possible that honokiol inhibits DENV replication by interfering with the expression of DENV NS1/NS3, and consequently suppressing the synthesis of viral dsRNA through mechanisms that require future investigation. Interestingly, it was reported that patients with DENV-2 infections experienced more severe disease than those infected with other serotypes [52]. Higher titers of DENV present in the blood during the viremic phase had also been linked with increased severity of dengue [52]. Therefore, viral load reduction by the use of effective antiviral agent, such as honokiol, may conceivably decrease the chance of severe dengue complications.

Pre-treatment of honokiol did not reveal to have effects on DENV-2 infection (Figure 4). This may indicates that treatment of honokiol prior to DENV infection did not alter any host cell-related mechanisms concerning the attachment of DENV. However, honokiol did interfere with the endocytotic process of DENV-2 (Figure 5). Subsequent to receptor-mediated endocytosis, many enveloped viruses use virus-endosome fusion to transport viral genome into the cytosol of the host cell. DENV is reported to fuse with late endosomes, while the activation of fusogenic DENV E protein is triggered at a pH-dependent characteristic for early endosomes [25,53,54]. The interaction of DENV E protein and the early endosome is an early event of DENV entry during virus-endosome fusion [25]. In our study, the presence of honokiol (10 and 20 µM) after DENV entry substantially suppressed the up-regulation of early endosomes. Moreover, the treatment of honokiol abrogated the co-localization of DENV E protein and the early endosome (Figure 5). This observation suggests that honokiol can interfere with the process of DENV-endosome fusion by mechanisms yet to be determined. A fluorescence resonance energy transfer-based single-virus tracking assay [55] was performed to further confirm the interference of honokiol on DENV entry. Our preliminary results also supported that honokiol interferes with the endocytosis of DENV during its entry (unpublished observation). Altogether, these results indicate that honokiol can interfere with the endocytotic process of DENV-2, which may reduce the release of viral RNA into cytoplasm and inhibit the subsequent viral gene expression and viral genome replication in host cells.

In a previous study, HepG2 cell was demonstrated to be more permissive for DENV infection and replication in the G2 phase [28], suggesting that cell cycle may be a mediator of cell permissiveness for DENV. Honokiol has been demonstrated to induce G0-G1 cell cycle arrest and eventually lead to apoptosis in cancer cells [27]. The effect of honokiol on the cell cycle regulation was studied. It was found that treatment of honokiol at MNTD did not have a discernible influence on the cell cycle of BHK cells, and the effect on the Huh7 cells was fairly limited (~10%, Figure 6). At the MNTD of honokiol treatment, the inhibition of DENV-2 replication was very prominent (>90% reduction, Figure 2). These results indicate that honokiol is not likely to suppress the DENV replication by modulating cell cycles in BHK and Huh7 cells.

NF-κB is an essential molecule in the induction of interferon (IFN)-β and cytokines in the toll-like receptor (TLR) signaling pathway [29,30], and these factors play vital roles in innate immunity against DENV infection [56]. However, a recent study has indicated that NF-κB activation triggered by TLR ligands is preferentially blocked by DENV-2 PL046 infection [31]. In this study, the honokiol-mediated DENV inhibition through modulation of NF-κB activation in Huh7 cells was investigated. It was found that neither infection of DENV-2 nor honokiol treatment induced NF-κB activation in Huh7 cells *in vitro* (Figure 7A). In a previous study, it was also observed that Huh7 cells infected with DENV were incapable of inducing the activation of NF-κB [32]. Rather than promoting, honokiol was reported to inhibit NF-κB activation in several cell types [33,34]. Thus, NF-κB activation is dispensable for the inhibitory effect of honokiol on DENV infection in this model. In virus-infected cells, the onset of IFN-α/β response occurs on viral entry and release or synthesis of viral components [57]. Several transcription factors, including IFN regulatory factor (IRF)-3, IRF-7, NF-κB, and activating transcription factor 2 (ATF2)/c-Jun are being activated and trigger the expression of IFN-α/β [58]. The released IFN-α/β elicits the antiviral response of the infected and neighboring cells. In this study, neither the expression of the IFN-β nor the activation of IRF-3 (an upstream regulator of IFN-β) was altered in Huh7 cells after DENV infection or honokiol treatments (Figure 7B and Figure S2). The low/no responsiveness of the Huh7 cell line in terms of NF-κB activation and IFN-β expression to DENV infection or poly(I:C) treatment was reported due to the intrinsic defect in TLR3 expression [42]. Collectively, these results suggest that honokiol can still exert its anti-DENV response effectively in Huh7 cells without regulating the NF-κB or IFN-βpathways.

In summary, we demonstrated that honokiol, a lignan biphenol extract from the *Magnolia* tree, exhibits profound antiviral activity against DENV-2. Honokiol suppresses the replication of DENV-2 in infected cells *in vitro*. Treatment with honokiol inhibits the expression of DENV NS1, NS3, dsRNA, and interferes with the entry process of DENV. Honokiol stands as a potentially promising new therapeutic agent for anti-DENV chemotherapy.

## 4. Materials and Methods

### 4.1. Cell Lines, Virus, and Chemicals

Baby hamster kidney cells (BHK; BCRC60041; obtained from Bioresource Collection and Research Center, Taiwan) were cultured in Dulbecco's modified Eagle’s medium (DMEM) supplemented with 2% fetal bovine serum (HyClone, Waltham, MA, USA) at 37 °C with 5% CO_2_. Human hepatocarcinoma Huh7 cells were cultured in DMEM with 10% FBS and BHK-D2-Fluc-SGR-Neo-1cells were maintained in the same medium with additional 10 µg/mL G418. BHK-D2-Fluc-SGR-Neo-1 is a stably transfected BHK cell line harboring a luciferase-reporting DENV subgenomic replicon [21]. A clinical isolate of DENV-2 (strain PL046, GenBank accession: AJ968413.1) was propagated as described previously [59]. The PL046 DENV was used at the multiply of infection (MOI) of 0.1 and 1, respectively, to infect BHK and Huh7 cells in this study, unless otherwise noted. Honokiol (39,5-di-2-propenyl-1,19-biphenyl-2,49-diol) was obtained from BioVision (Milpitas, CA, USA). 12-O-tetradecanoylphorbol 13-acetate (PMA) was obtained from Sigma-Aldrich (St. Louis, MO, USA). Polyinosinic-polycytidylic acid (poly(I:C)) was purchased from Invivogen (San Diego, CA, USA). Honokiol was dissolved in dimethyl sulfoxide (DMSO) as a stock solution of 100 mM and further diluted in culture medium to appropriate final concentration when used, with the final content of DMSO not exceeding 0.5%. Mouse monoclonal antibody against DENV-2 NS1 (clone 206-35), NS3 (clone 9-9), and envelope E protein (clone 137-22) were constructed previously [60] and were purified from hybridoma culture supernatant using Montage Prosep-G kit (Millipore, Bedford, USA).

### 4.2. Cell Cytotoxicity Assay

Cell viability was determined using the standard MTT assay. Briefly, cells were seeded in 96-well plates at a density of 3 × 10^3^ cells/100 µL per well overnight and then treated with honokiol at various concentrations (final volume 200 µL per well after addition of honokiol) for the times indicated. At the end of treatments, 20 µL of 3-(4,5-dimethylthiazol-2-yl)-2,5-diphenyltetrazolium bromide (MTT; 2.5 mg/mL; Sigma-Aldrich, St. Louis, MO, USA) was added to the culture medium and incubation continued at 37 °C for 3 h. After incubation, the supernatant was removed and the formation of dark formazan was dissolved in DMSO and measured using a microplate reader at an absorption wavelength of 570 nm. The 50% cytotoxic concentration (CC_50_) was defined as the compound’s concentration (µM) required for the reduction of cell viability by 50%, which was calculated by regression analysis.

### 4.3. DENV Replicon Assay

BHK-D2-Fluc-SGR-Neo-1, a stably transfected BHK cell line harboring a luciferase-reporting DENV subgenomic replicon [21], was seeded in 96-well plates at a density of 5 × 10^3^ cells/100 µL per well overnight and then treated with honokiol at various concentrations for 24 h. The culture medium was removed and cells were rinsed two times with PBS. Cells were lysed with 100 µL of lysis buffer (Promega, Madison, WI, USA), and the luciferase activity was evaluated following the manufacturer’s protocol (Luciferase Assay System, Promega). The luciferase activity was measured by microplate reader at an absorption wavelength of 640 nm (EnSpire multimode plate reader, PerkinElmer, Waltham, MA, USA).

### 4.4. Viral Yield Reduction and Fluorescence Focus Formation Assay

The BHK and Huh7 cells were first infected with DENV-2 by incubation with medium containing the virus for 1 h. After infection, the medium was replaced with virus-free medium containing a different concentration of honokiol and incubated for 48 h. The supernatant were collected, diluted 10-fold serially with serum free medium, and added to a 96-well plate with confluent BHK cells. After 1 h of incubation, 100 µL of DMEM/FBS containing 0.8% methyl cellulose was added to each well and the cells were cultured at 37°C with 5% CO_2_ for 72 h. The resulting cells were washed with PBS, fixed with 3.7% formaldehyde/PBS, and then permeabilized with 0.1% Triton X-100/PBS. The anti-DENV-2 NS1 antibody 206-35 was added and incubated for 1.5 h. The cells were then washed and incubated with secondary HRP-conjugated goat anti-mouse IgG antibody (1:1000, GeneTex) for 1.5 h at room temperature. After extensive washes, tyramide-fluorescein [61] was added to each well and the fluorescent foci were analyzed by the Typhoon FLA 9000 Biomolecular Imager (GE Healthcare, Pittsburgh, PA, USA).

### 4.5. Immunofluorescence Staining

The following procedure was used for immunofluorescence staining in this study. After treatment, cells were washed twice in PBS and followed by fixation with 3.7% formaldehyde/PBS and permeabilization with 0.1% Triton X-100/PBS, except for dsRNA staining where the cells were fixed and permeabilized with ice-cold methanol for 15 min. The cells were incubated with primary antibody for 1.5 h at room temperature, and then washed and incubated with corresponding secondary antibody (1:5000) for 30 min at room temperature. Cell nuclei were stained with Hoechst 33258 (1 µg/mL) for 10 min. Immunostained cells were washed thoroughly with PBS and then examined by confocal microscopy or BD Pathway™ 435 high-content cell analyzers (BD Biosciences, Rockville, MD, USA). Antibodies against DENV-2 NS1 (clone 206-35), NS3 (clone 9-9), and envelope E protein (clone 137-22) [60], dsRNA (clone J2, English & Scientific Consulting, Bt, Szirák, Hungary), early endosome antigen-1 (EEA1, GeneTex, Irvine, CA, USA), IRF-3 (Abcam, Cambridge, UK) were used as the primary antibodies in these analyses. Anti-mouse IgG Alexa 488, anti-rabbit IgG Alexa 488, and anti-rabbit IgG Alexa 546 (Invitrogen, Eugene, Oregon), were used as secondary antibodies. For immunofluorescence assay of viral dsRNA, NS1 and NS3, cells were seeded in Cellcarrier-96well plates overnight and then infected with DENV-2 for 1 h. Honokiol was added at indicated concentration and incubated for 48 h. The resulting cells were immunofluorescence stained as described above. The images were acquired by BD Pathway™ 435 Bioimaging system and the percentage of viral dsRNA, NS1 or NS3 positive cells was analyzed using the Attovision software (BD Biosciences).

### 4.6. DENV Attachment Assay

To determine if pre-treatment of honokiol inhibits the attachment of DENV, BHK cells were seeded in 24-well plates at a density of 1 × 10^5^ cells per well overnight and then treated with vehicle or 10 µM honokiol for 24 and 48 h. After treatment, the cells were detached and incubated with DENV at MOI = 10 for 30 min at 4 °C. The neutralizing anti-DENV E antibody 137-22 was used as a control to block the specific DENV binding to host cells. After incubation, the cells were washed twice with PBS and then incubated with anti-DENV E antibody (137-22) for 30 min at 4 °C. The cells were then washed and incubated with anti-mouse IgG Alexa 488 and analyzed by flow cytometry (Cytomics FC500, Beckman Coulter, CA, USA).

### 4.7. Assay of DENV Endocytosis

Huh7 cells were first infected with DENV on ice for 1 h and then washed twice with PBS. Medium containing vehicle or honokiol was then added and the cells were incubated at 37 °C for 90 min. The cells were then fixed, stained with first and secondary antibody as described in the immunofluorescence staining section. DENV E (clone 137-22) and EEA1 antibody were used to detect DENV E protein and early endosome, respectively.

### 4.8. Cell Cycle Analysis

Cells were treated with honokiol for 48 h. After treatment, the cells were fixed in ice-cold 70% ethanol, washed with PBS, and then stained with propidium iodide/RNase A in PBS (Sigma-Aldrich). The cell cycle distribution of stained cells was analyzed by flow cytometry (Cytomics FC500, Beckman Coulter, CA, USA).

### 4.9. Promoter Activity Assay

The pGL4.32[*luc2P*/NF-κB-RE/Hygro] vector contains five copies of an NF-κB response element that drives transcription of the luciferase reporter gene (Promega). pGL4.74[*hRluc*/TK] is a Rellina luciferase expression vector that was used as a normalization control in this test (Promega). pLuc-IFN-β vector is an IFN-β promoter-driven luciferase reporter [62]. Huh7 cells were seeded in 24-well plates at a density of 5 × 10^4^ cells per well overnight and then transfected with pGL4.32[*luc2P*/NFκB-RE/Hygro] or pLuc-IFN-β vector, along with pGL4.74[*hRluc*/TK] vector. After 24 h, the transfected cells were then mock or infected with DENV (MOI = 5), and treated with chemicals (DMSO, honokiol, PMA, or poly (I:C)) for 48 h. Cells were then collected and lysed with 100 µL of lysis buffer (Promega), and the luciferase activity was evaluated following the manufacturer’s protocol (Luciferase Assay System, Promega).

### 4.10. Statistical Analysis

Differences between multiple groups were analyzed by one-way ANOVA with Dunnett’s method for pairwise comparisons. *p* < 0.05 was considered to be statistically significant.

## Supplementary Materials

Supplementary materials can be found at: http://www.mdpi.com/1999-4915/7/9/2852/s1.
