# Supplementary material for: Honokiol, a Lignan Biphenol Derived from the Magnolia Tree, Inhibits Dengue Virus Type 2 Infection
Source: Viruses. 2015 Sep 10;7(9):4894–910. doi: 10.3390/v7092852 (PMC4584296; doi:10.3390/v7092852)
Supplement: Supplementary file 1 [file viruses-07-02852-s001.pdf]

Supplementary Information

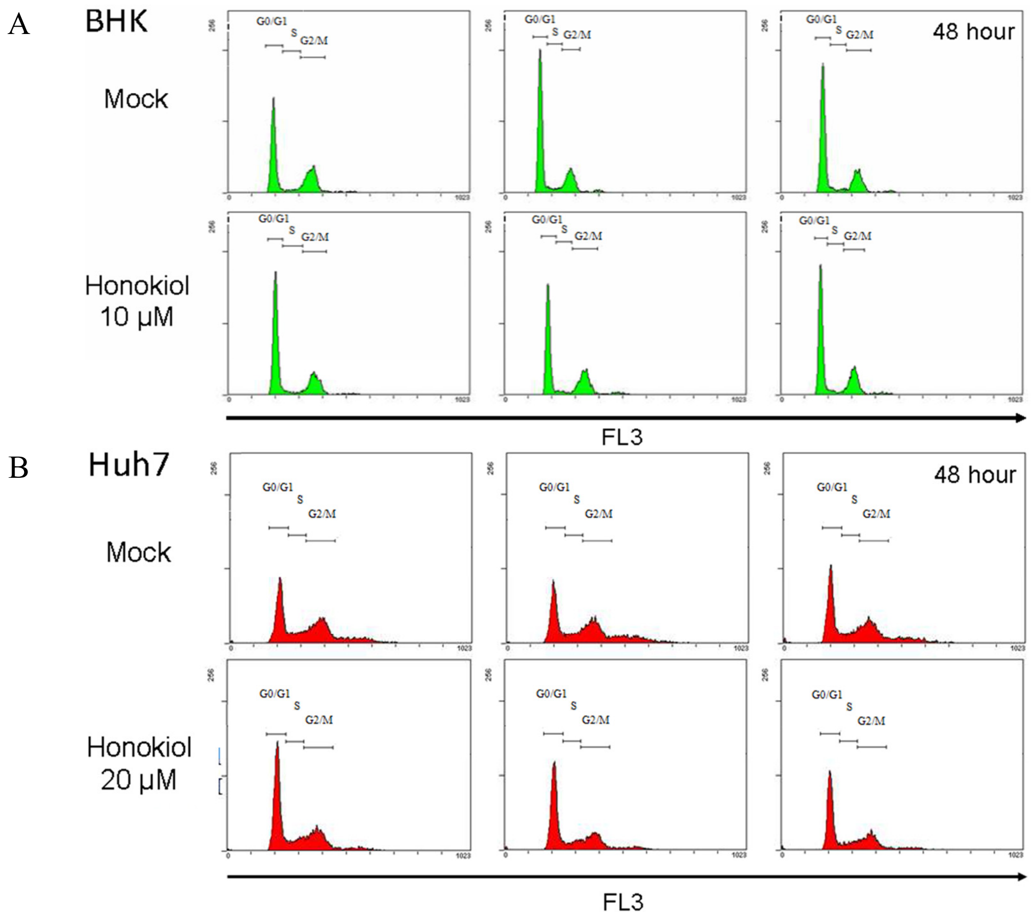

Figure S1. Cell cycle analysis of honokiol-treated BHK and Huh7 cells. (**A**) BHK, and (**B**) Huh7 cells were treated with honokiol for 48 h and the cell cycle distribution was analyzed by flow cytometry.

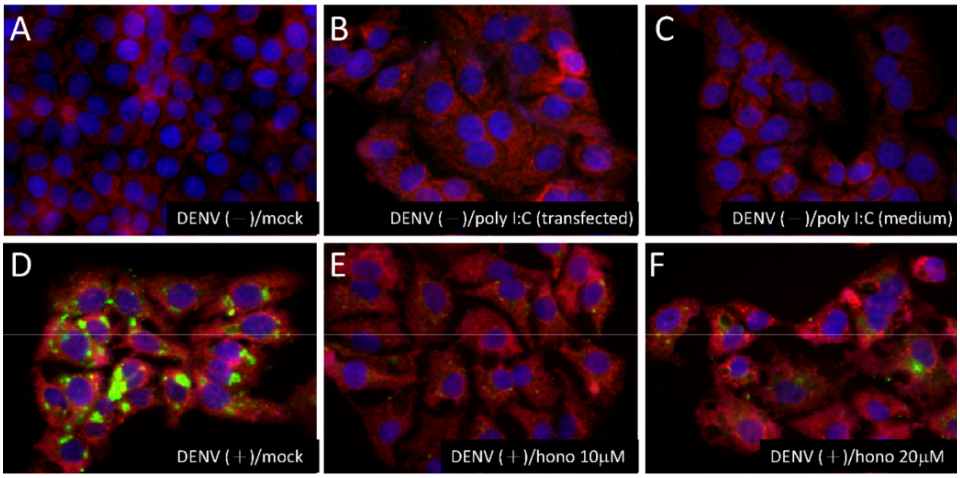

Figure S2. DENV infection and honokiol treatment does not induce the nuclear translocation of IRF-3. Uninfected Huh7 cells were (**A**) mock-treated, (**B**) transfected with ploy I:C (1.0 μg), or (**C**) treated with poly I:C (5 μg/mL) in medium for 48 h. DENV-infected Huh7 cells were (**D**) mock-treated or incubated with honokiol at (**E**) 10 μM and (**F**) 20 μM for 48 h. Immunofluorescence staining was conducted to detect the DENV E protein (green fluorescence) and the IRF-3 (red fluorescence). Cell nuclei were stained with Hoechst 33258.
